# Impact of prior vaccination on clinical outcomes of patients with COVID-19

**DOI:** 10.1080/22221751.2022.2069516

**Published:** 2022-05-23

**Authors:** Woo Jung Seo, Jiyeon Kang, Hyung Koo Kang, So Hee Park, Hyeon-Kyoung Koo, Hye Kyeong Park, Sung-Soon Lee, Je Eun Song, Yee Gyung Kwak, Jieun Kang

**Affiliations:** a Division of Pulmonary and Critical Care Medicine, Department of Internal Medicine, Inje University College of Medicine, Ilsan Paik Hospital, Goyang, Republic of Korea; b Division of Infectious Diseases, Department of Internal Medicine, Inje University College of Medicine, Ilsan Paik Hospital, Goyang, Republic of Korea

**Keywords:** SARS-CoV-2, COVID-19, vaccination, pneumonia, clinical outcome

## Abstract

Fully vaccinated people remain at risk of Coronavirus Disease 2019 (COVID-19). We examined association between prior vaccination and clinical outcomes in patients with COVID-19. Overall, 387 patients with mild-to-severe COVID-19 were enrolled. Patients were considered fully vaccinated at least 14, 7, and 14 days after receiving the second dose of ChAdOx1 nCoV-19 or mRNA-1273, second dose of BNT162b2, or single dose of Ad26.COV2.S, respectively. The primary outcomes (risk of pneumonia, requirement of supplemental oxygen, and progression to respiratory failure) were compared between vaccinated and unvaccinated patients. Logistic regression analysis was performed to identify factors associated with the outcomes. There were 204 and 183 patients in the vaccinated and unvaccinated groups, respectively. The vaccinated group was significantly older and had more comorbidities than the unvaccinated group. Patients in the unvaccinated group were significantly more likely to develop pneumonia (65.6% vs. 36.8%) or require supplemental oxygen (29.0 vs. 15.7%) than the vaccinated group. The vaccinated group had a significantly shorter time from symptom onset to hospital discharge than the unvaccinated group (10 vs. 11 days; *p*<0.001). The proportion of patients who progressed to respiratory failure did not differ significantly between groups. In multivariable analyses, vaccination was associated with an approximately 70% and 82% lower likelihood of pneumonia and supplemental oxygen requirement, respectively. Being vaccinated was associated with a significantly lower risk of pneumonia and severe disease when breakthrough infection developed. Our findings support continuous efforts to increase vaccine coverage in populations.

## Introduction

The Coronavirus Disease 2019 (COVID-19) pandemic, caused by the Severe Acute Respiratory Syndrome Coronavirus 2 (SARS-CoV-2), has been a significant global health threat for the past couple of years [1]. As of 26 January 2022, there were over 350 million confirmed cases of COVID-19 [2]. Several risk factors have been suggested for COVID-19 infection such as older age, presence of comorbidities, and prior pneumonia [3,4]. A recent Greek study showed a high prevalence of risk factors in the community [4] when various age groups were surveyed via web-based questionnaires, and 81.2% of 2000 respondents reported having underlying medical conditions that could lead to severe COVID-19. Due to the high morbidity and mortality of COVID-19 [5,6], along with several quarantine regulations, the importance of vaccination is being emphasized [7]. Following the demonstration of effective prevention of COVID-19 by several vaccines in phase-3 trials, they have been approved [8-11]. These include mRNA vaccines with 94-95% efficacy in preventing SARS-CoV-2 infection [9,10].

However, even fully vaccinated people remain at risk of COVID-19 (breakthrough infections) [12,13]. Causes of breakthrough infections include incomplete vaccine efficacy, decline in protective effects over time, differences in individual immune responses after vaccination, and emergence of variants [14]. With increases in population vaccine coverage, the number of breakthrough infection cases is expected to rise [15]. To date, little is known about how prior vaccination affects the clinical outcomes and prognosis of breakthrough infections.

The clinical presentation of COVID-19 varies, ranging from pauci-symptomatic infection to the severe forms of respiratory failure [16,17]. Pneumonia or oxygen demand in COVID-19 can be an alarming signal of possible worsening respiratory status and may burden limited medical resources. Furthermore, long-term sequelae may persist even in survivors [18-20]. To determine the impact of prior vaccination on breakthrough infection, we compared the clinical outcomes of vaccinated and unvaccinated patients hospitalized for COVID-19 and examined the association between prior vaccination and patient outcomes.

## Materials and methods

### Study patients

This was a retrospective study involving patients with a confirmed diagnosis of COVID-19 admitted to Ilsan Paik Hospital, Republic of Korea. Between 1 September and 31 December 2021, 389 patient hospitalizations were identified with COVID-19. The two patients who were transferred from other hospitals for post-acute care were excluded from the analysis, leaving 387 in the current study.

Patients were considered fully vaccinated at least 14 days after receipt of the second dose of ChAdOx1 nCoV-19 (Oxford/AstraZeneca, Cambridge, UK) or mRNA-1273 (Moderna Inc., Cambridge, USA), at least 7 days after the second dose of BNT162b2 (Pfizer/bioNTech, New York, NY, USA and Mainz, Germany), or at least 14 days after receiving a single dose of Ad26.COV2.S (Janssen-Cilag International NV, Beerse, Belgium). Vaccination status was classified according to vaccine receipt before the date of symptom onset in symptomatic patients and the date of diagnosis in asymptomatic patients. The study protocol was approved by the Institutional Review Board of Ilsan Paik Hospital (No. 2022-01-025). The need for informed consent was waived owing to the retrospective nature of the study.

### COVID-19 severity classification

According to the Korea Disease Control and Prevention Agency guidelines, all patients with COVID-19 were classified by their initial level of severity to determine who required hospitalization and who could be treated at home or in residential treatment centres. Symptomatic patients were categorized into one of four groups (mild, moderate, severe, and critical illness) by the city and provincial patient management teams (Supplementary Table 1). Our hospital is designated to treat patients with mild, moderate, or severe COVID-19. Therefore, patients with critical illnesses (i.e. respiratory failure, septic shock, and/or multiple organ dysfunction) at the time of diagnosis were not included in the analysis.

### Treatment and discharge

Treatment was provided in accordance with the revised Korean Society of Infectious Diseases/National Evidence-based Healthcare Collaborating Agency guidelines on the treatment of patients with COVID-19 [21]. Remdesivir was administered to patients who required supplemental oxygen but no invasive ventilation or extracorporeal membrane oxygenation. Corticosteroid administration was considered for patients with severe or critical disease. For patients with rapidly increased oxygen requirement, tocilizumab (interleukin-6 inhibitor) was added. Regdanvimab, a recombinant human monoclonal antibody against SARS-CoV-2, was considered in high-risk patients with mild-to-moderate disease who developed symptoms within 7 days before drug administration and who do not require supplemental oxygen.

Due to quarantine issues, the Korean government established the following criteria to be met by the patients before discharge: 1) improvements in clinical symptoms and no fever for at least 24 h, and 2) discharge after at least 10 days from the onset of symptoms. Asymptomatic patients were discharged if they did not show any clinical symptoms for 10 days after confirmation. When the admitted patients progressed to a critical condition (respiratory failure, septic shock, and/or multiple organ dysfunction), they were transferred to a hospital designated for critical care.

### Data collection

Baseline characteristics, including age, sex, body mass index (BMI), comorbidities, and details of COVID-19 vaccination, including dates and vaccine products, were obtained from the electronic medical records. The diagnosis date, presence of symptoms at hospital admission, date of symptom onset, admission and discharge dates, and cycle threshold (Ct) values of the RdRp/E/N genes from reverse transcription (RT)-polymerase chain reaction (PCR) were also recorded. All the patients underwent chest radiography for radiological evaluation. Some patients underwent chest computed tomography (CT), which was determined by the attending physician.

### Outcomes

The primary outcomes were the risks of pneumonia, requirement of supplemental oxygen, and progression to respiratory failure due to COVID-19. Pneumonia was defined as radiological evidence of pulmonary infiltrates on chest radiography or chest CT scan. The requirement of supplemental oxygen was determined when oxygen saturation was less than 94% on room air at sea level. Respiratory failure was defined as the requirement for oxygen supply via a high-flow nasal cannula, mechanical ventilation, or extracorporeal membrane oxygenation.

### Statistical analysis

Data are presented as mean ± standard deviation or median (interquartile range [IQR]) for continuous parameters and number (percentage) for categorical variables. Chi-squared and Fisher's exact tests were used to compare categorical variables, whereas for continuous variables, the t-test and Mann–Whitney U test were used for normally and non-normally distributed data, respectively. The probability of respiratory failure-free survival was assessed using Kaplan-Meier survival analysis, in which statistical significance was based on a log-rank test. Logistic regression analysis was used to determine the risk factors for pneumonia, requirement of supplemental oxygen, and progression to respiratory failure. Effects of the time interval from the second vaccination dose to admission on respiratory outcomes were also assessed using logistic regression analysis. Variables with *p* <0.1 in the unadjusted analysis were entered into the multivariable models and analysed using the backward LR or enter method. Sensitivity analyses were performed excluding patients who only received the first dose in a two-dose series, those who failed to meet the criteria for full vaccination due to a short interval between the receipt of a second dose and the development of COVID-19, and those who received a booster dose. All *p*-values were two-tailed, and *p* <0.05 was considered statistically significant. Data were analysed using IBM SPSS Statistics for Windows, version 25.0; (IBM Corp., Armonk, N.Y., USA).

## Result

### Baseline characteristics of the study patients

According to the vaccination status, 204 (52.7%) and 183 (47.3%) patients were in the vaccinated and unvaccinated group, respectively. In the vaccinated group, the median interval from the receipt of a second dose until onset of symptoms was 100.0 (IQR, 70.0–136.3) days whereas the median time from the second dose to hospital admission date was 106.5 (IQR, 73.5–141.3) days.

Table 1 shows the baseline characteristics of the two groups. In the vaccinated and unvaccinated groups, mean ages were 66.8 and 47.8 years, and men accounted for 50.5% and 49.2%, respectively. Patients in the vaccinated group were significantly older and more frequently had hypertension and chronic lung disease than those in the unvaccinated group. Sex, BMI, and the lowest Ct value by RT–PCR did not differ significantly between the groups.

**Table 1.** Baseline characteristics of patients according to the vaccination status

	Vaccinated (n = 204)	Unvaccinated (n = 183)	*p*-value
Age, y	66.8 ± 14.3	47.8 ± 16.8	<0.001
Age category, y			<0.001
18–49	27 (13.2)	101 (55.2)	
50–64	49 (24.0)	52 (28.4)	
≥65	128 (62.7)	30 (16.4)	
Sex			0.839
Male	103 (50.5)	90 (49.2)	
Female	101 (49.5)	93 (50.8)	
BMI, kg/m^2^	25.2 ± 3.8	25.6 ± 5.0	0.357
PCR Gene Ct value	17.2 [13.8;21.2]	17.6 [14.7;21.4]	0.445
Co-morbidities			
Hypertension	108 (52.9)	43 (23.5)	<0.001
Diabetes	46 (22.5)	26 (14.2)	0.037
Cardiovascular disease	35 (17.2)	26 (14.2)	0.486
Chronic lung disease	17 (8.3)	6 (3.3)	0.051
Chronic kidney disease	10 (4.9)	7 (3.8)	0.630
Chronic liver disease	3 (1.5)	8 (4.4)	0.125
Solid organ transplantation	3 (1.5)	2 (1.1)	>0.999
Rheumatic disorders	3 (1.5)	8 (4.4)	0.125
Cancer	16 (7.8)	11 (6.0)	0.552
Obesity (BMI>30)	31 (15.2)	31 (16.9)	0.679

Data are presented as number (%), median [interquartile range], or mean ± standard deviation.

Abbreviations: BMI, body mass index; PCR, polymerase chain reaction; Ct, cycle threshold.

### Types of vaccine administered

In the vaccinated group, the most commonly administered vaccine product was ChAdOx1 nCoV-19 (49.0%), followed by BNT162b2 (48.0%) (Table 2). Four patients who received ChAdOx1 nCoV-19 as the first dose received BNT162b2 as the second dose. Booster doses were administered to 13 patients: 11 received BNT162b2, and 2 received mRNA-1273.

**Table 2.** Types of vaccine product administered to the vaccinated group (n = 204)

	First dose	Second dose	Booster dose
ChAdOx1 nCoV-19	104 (51.0)	100 (49.0)	–
BNT162b2	94 (46.1)	98 (48.0)	11 (5.4)
mRNA-1273	1 (0.5)	1 (0.5)	2 (1.0)
Ad26.COV2.S	5 (2.5)		–

Data are presented as number (%).

### Proportion of breakthrough infection by month

**Figure 1.**
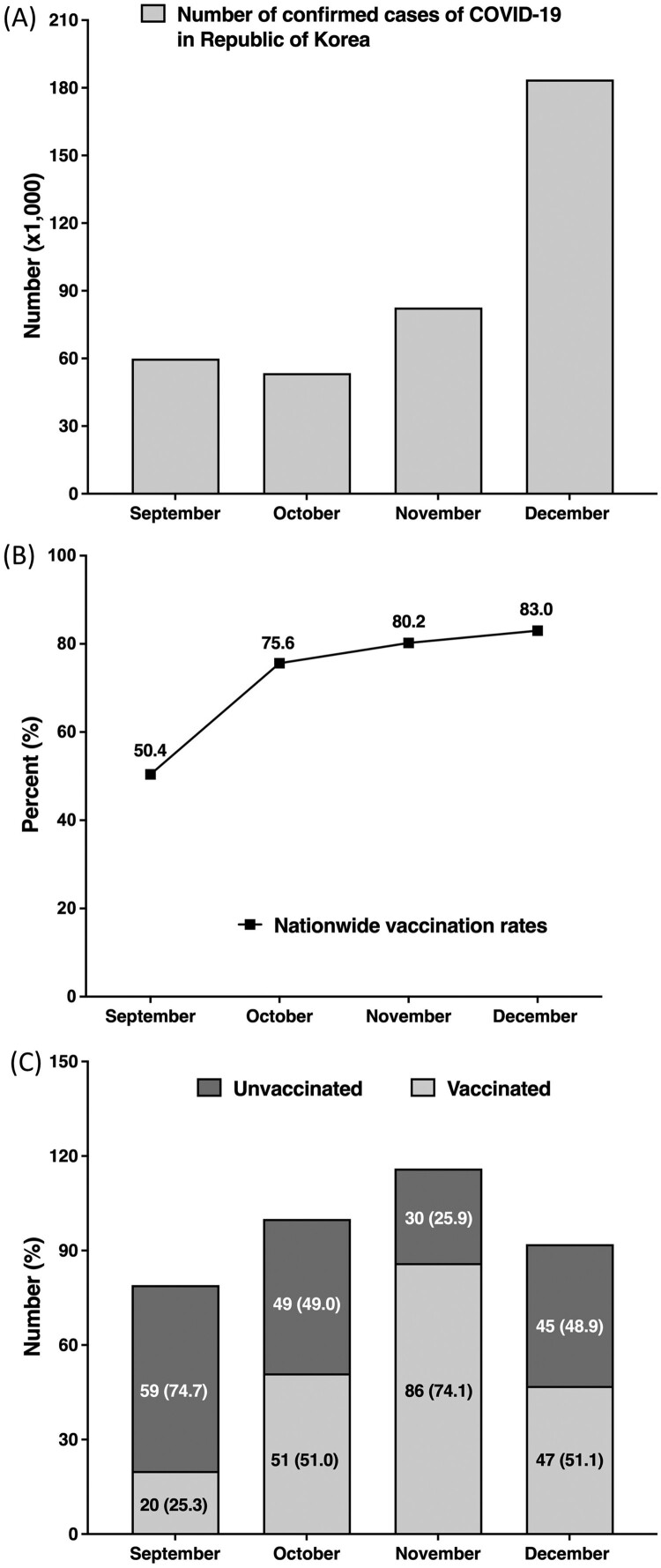
Nationwide trends in COVID-19 cases, vaccination rates, and proportion of breakthrough infections among study patients (a) Cumulative number of confirmed COVID-19 cases in Republic of Korea during the study period. (b) Trends in vaccination rates in Republic of Korea during the study period. (c) Relative proportion of vaccinated and unvaccinated patients in the study patients. Abbreviations: COVID-19, coronavirus disease 2019

Figure 1(A) and (B) show the number of COVID-19 confirmed cases and cumulative percentages of the fully vaccinated nationwide population, monthly, respectively. The vaccination rate of 50.4% in Republic of Korea at the end of September had increased to 83.0% by the end of December. Figure 1(C) shows the number of patients admitted to our hospital per month, relative proportion of vaccinated and unvaccinated patients, and cumulative rate of breakthrough infections. As the number of vaccinated individuals increased, the proportion of breakthrough infections among the study patients tended to increase.

### Initial presentation of covid-19 and clinical outcomes

**Figure 2.**
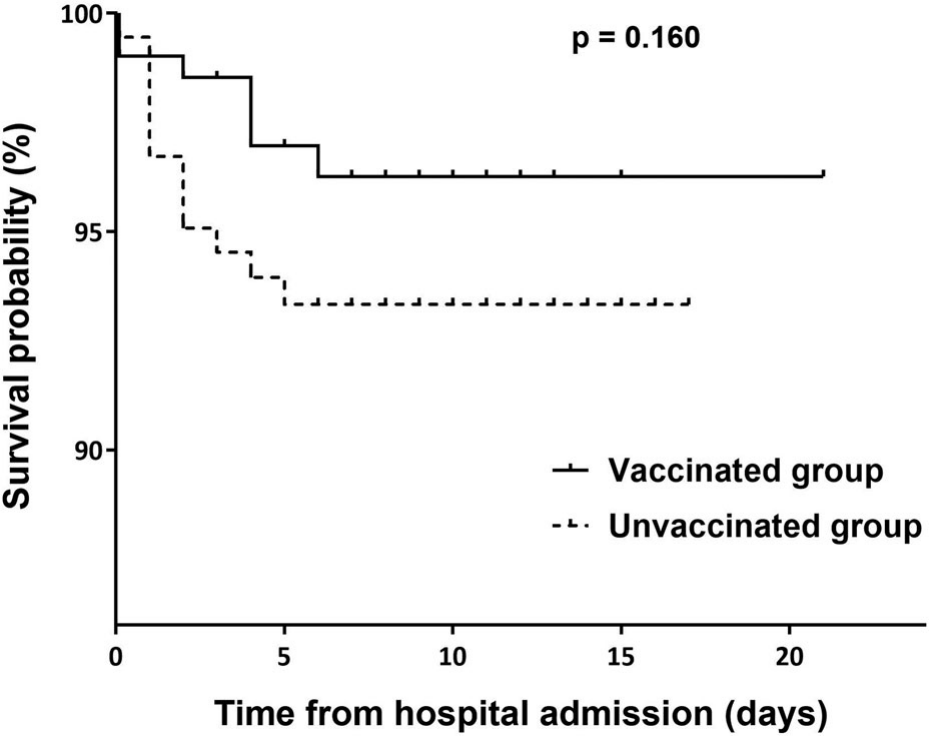
Kaplan-Meier curves of the respiratory failure-free survival by vaccination status. Kaplan-Meier survival curves of the probability of respiratory failure-free survival from hospital admission did not show a significant difference between the vaccinated and unvaccinated groups.

Table 3 shows the initial presentations and outcomes of COVID-19 in the vaccinated and unvaccinated patients. The unvaccinated group had a significantly higher proportion of patients with symptomatic infection (93.4% vs. 86.3%) and hypoxia at the time of hospitalization (17.5% vs. 9.8%) than the vaccinated group. Pneumonia was more frequent in the unvaccinated group than in the vaccinated group (65.6% vs. 36.8%). Patients in the unvaccinated group were significantly more likely to require supplemental oxygen than those in the vaccinated group (29.0% vs. 15.7%; *p* = 0.002) and to receive remdesivir (27.9% vs. 16.7%; *p* = 0.010). Dexamethasone was also administered more frequently in the unvaccinated group than in the vaccinated group (33.9% vs. 22.1%; *p* = 0.012). The vaccinated group had a significantly shorter time from symptom onset to hospital discharge than the unvaccinated group (10 vs. 11 days; *p*<0.001). The proportion of patients who progressed to respiratory failure was non-significantly higher in the unvaccinated group than in the vaccinated group. Kaplan-Meier analysis demonstrated no significant difference in respiratory failure-free survival between the vaccinated and unvaccinated groups (Figure 2). The clinical course according to the vaccine type is described in Supplementary Table 2.

**Table 3.** COVID-19 presentation and clinical course according to the vaccination status

	Vaccinated (n = 204)	Unvaccinated (n = 183)	*p*-value
Asymptomatic infection, (%)	28 (13.7)	12 (6.6)	0.029
Hypoxia* at admission, (%)	20 (9.8)	32 (17.5)	0.036
Pneumonia, (%)	75 (36.8)	120 (65.6)	<0.001
Supplemental oxygen requirement	32 (15.7)	53 (29.0)	0.002
Respiratory failure	7 (3.4)	12 (6.6)	0.166
Transfer, (%)	7 (3.4)	11 (6.0)	0.239
Treatment, (%)			
Remdesivir	34 (16.7)	51 (27.9)	0.010
Corticosteroid	45 (22.1)	62 (33.9)	0.012
Tocilizumab	3 (1.5)	6 (3.3)	0.317
Regdanvimab	130 (63.7)	85 (46.4)	0.001
Duration of oxygen supplementation, days	3.0 [2.0;4.8]	4.0 [2.0;6.0]	0.344
Time from symptom onset to discharge, days	10 [9;11]	11 [10;14]	<0.001

Data are presented as numbers (%) or medians [interquartile ranges].

*Hypoxia was defined as oxygen saturation <94% on room air at sea level.

Abbreviation: COVID-19, coronavirus disease 2019.

### Factors associated with covid-19 pneumonia

Various clinical variables were included in the logistic regression model to identify the risk factors for developing COVID-19 pneumonia (Table 4). In the unadjusted and multivariable analyses, only prior vaccination was significantly associated with a lower risk of COVID-19 pneumonia (adjusted odds ratio [OR], 0.305; 95% confidence interval [CI], 0.197–0.472; *p* <0.001).

**Table 4.** Factors associated with COVID-19 pneumonia

	Unadjusted OR (95% CI)	*p*-value	Adjusted OR (95% CI)	*p*-value
Age	1.004 (0.993–1.015)	0.464		
Male sex	1.269 (0.851–1.891)	0.242		
BMI	1.001 (0.956–1.048)	0.975		
PCR Ct value	0.996 (0.961–1.032)	0.839		
Hypertension	0.703 (0.467–1.060)	0.092	1.005 (0.643–1.571)	0.982
Diabetes	1.482 (0.883–2.488)	0.136		
Cardiovascular disease	1.118 (0.646–1.935)	0.692		
Chronic lung disease	1.079 (0.464–2.508)	0.860		
Chronic kidney disease	1.429 (0.532–3.834)	0.479		
Chronic liver disease	1.750 (0.504–6.078)	0.378		
Solid organ transplantation	1.484 (0.245–8.984)	0.667		
Rheumatic disorders	0.553 (0.159–1.922)	0.352		
Cancer	0.658 (0.297–1.456)	0.301		
Obesity (BMI>30)	0.925 (0.536–1.597)	0.781		
Prior vaccination	0.305 (0.201–0.463)	<0.001	0.305 (0.197–0.472)	<0.001

Abbreviations: COVID-19, coronavirus disease 2019; OR, odds ratio; CI, confidence interval; BMI, body mass index; PCR, polymerase chain reaction; Ct, cycle threshold.

### Factors associated with supplemental oxygen requirement

Factors associated with the requirement for oxygen supplementation in the unadjusted analysis included older age, presence of diabetes, and prior vaccination (Table 5). In multivariable analysis, prior vaccination was associated with an approximately 82% lower likelihood of supplemental oxygen requirement (adjusted OR, 0.184; 95% CI, 0.098–0.345; *p*<0.001).

**Table 5.** Factors associated with supplemental oxygen requirement

	Unadjusted OR (95% CI)	*p*-value	Adjusted OR (95% CI)	*p*-value
Age	1.020 (1.005–1.034)	0.007	1.046 (1.026–1.066)	<0.001
Male sex	1.102 (0.681–1.784)	0.693		
BMI	1.033 (0.979–1.091)	0.238		
PCR Ct value	1.005 (0.963–1.049)	0.826		
Hypertension	1.195 (0.733–1.948)	0.476		
Diabetes	1.760 (0.993–3.120)	0.053	1.581 (0.855–2.922)	0.144
Cardiovascular disease	1.189 (0.626–2.259)	0.596		
Chronic lung disease	0.736 (0.243–2.223)	0.586		
Chronic kidney disease	1.098 (0.349–3.458)	0.873		
Chronic liver disease	1.345 (0.349–5.183)	0.667		
Solid organ transplantation	0.887 (0.098–8.042)	0.915		
Rheumatic disorders	0.348 (0.044–2.755)	0.317		
Cancer	0.795 (0.292–2.167)	0.655		
Obesity (BMI>30)	1.440 (0.773–2.683)	0.250		
Prior vaccination	0.456 (0.278–0.748)	0.002	0.184 (0.098–0.345)	<0.001

Abbreviations: OR, odds ratio; CI, confidence interval; BMI, body mass index; PCR, polymerase chain reaction; Ct, cycle threshold.

### Factors associated with respiratory failure due to covid-19

Table 6 shows the predictors of respiratory failure in patients with COVID-19. In the unadjusted analysis, various comorbidities, such as hypertension, diabetes, cardiovascular disease, and chronic kidney disease, were associated with progression to respiratory failure, but vaccination was not a predictor. In the multivariate model, the presence of cardiovascular disease (adjusted OR, 3.188; 95% CI, 1.121–9.064; *p* = 0.030) was significantly associated with respiratory failure.

**Table 6.** Factors associated with progression to respiratory failure

	Unadjusted OR (95% CI)	*p*-value	Adjusted OR (95% CI)	*p*-value
Age	1.031 (1.001–1.063)	0.043		
Male sex	0.900 (0.357–2.266)	0.823		
BMI	1.056 (0.963–1.159)	0.727		
PCR Ct value	0.915 (0.830–1.009)	0.074	0.919 (0.830–1.018)	0.104
Hypertension	2.824 (1.086–7.344)	0.033		
Diabetes	2.719 (1.031–7.172)	0.043		
Cardiovascular disease	3.025 (1.074–8.518)	0.036	3.188 (1.121–9.064)	0.030
Chronic liver disease	0.503 (0.061–4.145)	0.523		
Chronic kidney disease	4.741 (1.237–18.175)	0.023		
Rheumatic disorders	1.989 (0.241–16.396)	0.523		
Obesity (BMI>30)	0.320 (0.042–2.470)	0.275		
Prior vaccination	0.506 (0.195–1.315)	0.162		

Multivariable logistic regression model was performed using the backward LR method.

Abbreviations: OR, odds ratio; CI, confidence interval; BMI, body mass index; PCR, polymerase chain reaction; Ct, cycle threshold.

### Effects of time from vaccination to hospital admission on the risk of respiratory outcomes

The time interval from the second vaccination dose to hospital admission according to the vaccine type is illustrated in Supplementary Figure 1. In the vaccinated group, increased time after vaccination was significantly associated with a higher risk of supplemental oxygen requirement (OR, 1.009; 95% CI, 1.001–1.017; *p* = 0.022) although no significant association was found with pneumonia or respiratory failure (Supplementary Table 3).

### Sensitivity analyses

There were 190 and 140 patients in the vaccinated and unvaccinated groups, respectively. The vaccinated group was significantly older and had a higher proportion of patients with hypertension (Supplementary Table 4). Among these patients, the vaccinated group had more asymptomatic infections (12.1% vs. 5.0%), less hypoxia at hospitalization (8.9% vs. 20.7%), less frequent pneumonia occurrences (36.3% vs. 71.4%), and supplemental oxygen requirement (15.3% vs. 35.0%) (Supplementary Table 5). The median interval from symptom onset to discharge was significantly shorter (10 vs. 12 days; *p* <0.001). In multivariable regression models, prior vaccination was significantly associated with a lower likelihood of pneumonia (adjusted OR, 0.230; 95% CI, 0.141–0.376; *p* <0.001) and supplemental oxygen requirement (adjusted OR, 0.130; 95% CI, 0.066–0.256; *p* <0.001).

## Discussion

In this study involving 387 patients with mild-to-severe COVID-19, vaccinated patients showed favourable outcomes despite their significantly older age and more frequent comorbidities. The initial viral load (represented by PCR Ct values) appeared similar between the vaccinated and unvaccinated groups, but in fully vaccinated patients, a significantly lower risk of pneumonia as well as supplemental oxygen requirement due to COVID-19 occurred.

In terms of vaccine effectiveness, studies have primarily focused on the prevention of symptomatic infection and hospitalization; not only phase 3 clinical trials, but also real-world studies have demonstrated that currently available vaccines are effective in preventing SARS-CoV-2 infection [22-24]. However, breakthrough cases have been detected and vaccine effectiveness against SARS-CoV-2 infection has been reported to wane over time [25,26]. In a recent study conducted in a large healthcare system assessing over 3 million patients, Tartof et al. reported that the protective effect of BNT162b2 declined to 47% after 5 months of vaccination [27]. Our study presents similar finding, with a median interval from vaccination to hospitalization of 106.5 days. It is important to note that fully vaccinated individuals remain at risk for COVID-19 and further research to clarify the clinical course of breakthrough infections is of clinical value.

According to the results of our study, vaccinated patients were significantly more likely to be asymptomatic. In addition, vaccinated patients had a significantly lower risk of pneumonia or supplemental oxygen requirement than unvaccinated patients. The median time from symptom onset to hospital discharge was significantly shorter in the vaccinated group. These findings support current recommendations for vaccination. Pneumonia is a serious complication of the SARS-CoV-2 infection. A proportion of patients deteriorate into acute respiratory failure with poor survival [28], and even after recovery, symptoms may linger [29,30]. More importantly, there have been reports of lung fibrosis as a post-COVID complication, and severity of illness as a risk factor [31]. One study found that the duration of oxygen supplementation was associated with abnormal findings of the lung function and chest CT during the follow-up [32]. Therefore, even in patients in whom vaccination fails to prevent infection, it provides benefits by reducing the incidence and severity of COVID-19 pneumonia.

Data on the mechanisms by which vaccination reduces infection severity are lacking. One plausible reason for this is the lower viral load in vaccinated patients. A previous study in Israel demonstrated that the mean Ct value of SARS-CoV-2 at RT–PCR, a surrogate for viral load, was significantly lower in fully vaccinated patients than in unvaccinated patients [33]. This alone may be an insufficient explanation because there was no significant difference in median Ct values between the vaccinated and unvaccinated groups in our study. Another plausible explanation is the enhanced antibody response against SARS-CoV-2 in vaccinated individuals [34,35]. We did not measure the antibody concentrations in this study, and it is difficult to determine whether enhanced antibody response led to a lower risk of pneumonia and less disease severity in the vaccinated patients. Further research is needed to determine how immune response against SARS-CoV-2 differs between vaccinated and unvaccinated patients.

The proportion of patients who progressed to respiratory failure was lower in the vaccinated group than in the unvaccinated group, but the difference was not statistically significant. The number of patients with respiratory failure might have been too small to detect a significant difference. Another hypothesis that may explain the variations in clinical outcomes in the unvaccinated group is pre-existing immunity against SARS-CoV-2. Although uncommon, re-infections occur; some in the unvaccinated group might have had pre-existing immunity from unrealized prior infection, which could have prevented progression to respiratory failure.

The risk factors for respiratory failure in our study were similar to those previously reported [36]; older age; comorbid conditions (hypertension, diabetes, cardiovascular disease, and chronic kidney disease); and lower PCR Ct value were significantly associated with progression to respiratory failure in the unadjusted model. Meanwhile, a recent study in the US found that prior vaccination had a protective effect against respiratory failure or death [15]. The study analysed 1983 hospitalized patients with COVID-19 and found that vaccination was associated with an approximately one-third lower likelihood of progression to death or mechanical ventilation. That study included patients vaccinated with mRNA vaccines; thus, interpretation may be limited when applied to a population vaccinated with other types of vaccines (e.g. viral vector vaccines). Furthermore, the study might not have fully encompassed the Delta variant dominance, given the study period (from 11 March to 15 August 2021). Nevertheless, the study indicated the benefit of prior vaccination on the outcomes of breakthrough infections, which is in line with our findings.

Most of the study patients included in our analysis were immunocompetent, although some immunocompromised patients, including those with cancer and rheumatic disorders, and solid organ transplant recipients were included. The impact of vaccination on breakthrough infections may be different in individuals with chronic immunosuppression. Recent research has shown that patients with cancer who develop COVID-19 despite full vaccination are at a risk of critical outcomes [37]. This recent study included 1787 patients with cancer and COVID-19, of whom 131 were fully or partially vaccinated. Patients with active or progressive cancer had an approximately 6-fold higher risk of 30-day mortality. Meanwhile, in patients with systemic rheumatic disorders, vaccination provided better outcomes for breakthrough infections, especially in terms of hospitalization and death [38]. Subgroups with specific underlying diseases may differ in vaccine benefits in cases of breakthrough infections; therefore, further research is needed to elucidate this matter.

This study has some limitations that need to be addressed. First, there might have been selection bias in the study patients; more than half of the patients in our study were fully vaccinated. Elderly patients and those with comorbidities were prioritized for receiving vaccines in the early phases of the distribution process, in accordance with the government guidelines in Republic of Korea. These patients were also given priority in hospitalization rather than being treated at home or residential treatment centres because they were considered at high risk. This led to a relatively high proportion of patients who were fully vaccinated but were infected with SARS-CoV-2 in our study. Nevertheless, our finding that clinical outcomes were favourable in patients who were considered high-risk [39,40] supports the effectiveness of vaccination. Second, since genotyping of SARS-CoV-2 was not performed in our study patients, we did not have information on the variant type in each case, and the predominant variant among the study patients. Since the detection rate of the Delta variant accounted for more than 50% of the local cases in Republic of Korea by the end of July 2021 [41], the Delta variant may have been the predominant type in our study patients. Recently, the Omicron variant surge has been sweeping the globe, becoming the predominant variant type [42,43]. It is uncertain whether the study results could be applied as it is, in the face of the Omicron variant dominance. However, recent studies have revealed that booster immunization with mRNA vaccines significantly increases the neutralizing activity against Omicron [44,45]. Third, 13 (6.4%) patients in the vaccinated group received a booster dose after the second vaccine dose. The prognosis of breakthrough infection may differ slightly from the results of our study in the context of an increased rate of booster vaccination. Given the benefit of vaccination even without a booster dose found in our study, we believe that the effectiveness of vaccination in reducing the severity of COVID-19 in breakthrough infections, is likely to be the same.

In conclusion, we found that being vaccinated was associated with a lower risk of pneumonia and severe disease when breakthrough infections occur, although the vaccinated had more risk factors. Our findings support continuous efforts to increase vaccine coverage in populations.

## Supplementary Material

Supplemental Material

## Data Availability

Data are available upon reasonable request.

## References

[1] Pollard CA, Morran MP, Nestor-Kalinoski AL. The COVID-19 pandemic: a global health crisis. Physiol Genomics. 2020;52(11):549–557. 32991251 10.1152/physiolgenomics.00089.2020 PMC7686876

[2] World Health Organization. WHO coronavirus (COVID-19) dashboard. Available from: https://covid19.who.int/ (accessed 26 January 2022).

[3] Zhou F, Yu T, Du R, et al. Clinical course and risk factors for mortality of adult inpatients with COVID-19 in wuhan, China: a retrospective cohort study. Lancet. 2020;395(10229):1054–1062. 32171076 10.1016/S0140-6736(20)30566-3 PMC7270627

[4] Mouliou DS, Kotsiou OS, Gourgoulianis KI. Estimates of COVID-19 risk factors among social strata and predictors for a vulnerability to the infection. Int J Environ Res Public Health. 2021;18(16):8701. 34444450 10.3390/ijerph18168701 PMC8392732

[5] Matta S, Chopra KK, Arora VK. Morbidity and mortality trends of COVID 19 in top 10 countries. Indian J Tuberc. 2020;67(4s):S167–s172. 33308665 10.1016/j.ijtb.2020.09.031 PMC7543896

[6] Villar J, Ariff S, Gunier RB, et al. Maternal and neonatal morbidity and mortality among pregnant women with and without COVID-19 infection: The INTERCOVID multinational cohort study. JAMA Pediatr. 2021;175(8):817–826. 33885740 10.1001/jamapediatrics.2021.1050 PMC8063132

[7] World Health Organization. COVID-19 advice for the public: Getting vaccinated. Available from: https://www.who.int/emergencies/diseases/novel-coronavirus-2019/covid-19-vaccines/advice (accessed 25 January 2022).

[8] Falsey AR, Sobieszczyk ME, Hirsch I, et al. Phase 3 safety and efficacy of AZD1222 (ChAdOx1 nCoV-19) COVID-19 vaccine. N Engl J Med. 2021;385(25):2348–2360. 34587382 10.1056/NEJMoa2105290 PMC8522798

[9] Polack FP, Thomas SJ, Kitchin N, et al. Safety and efficacy of the BNT162b2 mRNA COVID-19 vaccine. N Engl J Med. 2020;383(27):2603–2615. 33301246 10.1056/NEJMoa2034577 PMC7745181

[10] Baden LR, El Sahly HM, Essink B, et al. Efficacy and safety of the mRNA-1273 SARS-CoV-2 vaccine. N Engl J Med. 2021;384(5):403–416. 33378609 10.1056/NEJMoa2035389 PMC7787219

[11] Sadoff J, Gray G, Vandebosch A, et al. Safety and efficacy of single-dose Ad26.COV2.S vaccine against COVID-19. N Engl J Med. 2021;384(23):2187–2201. 33882225 10.1056/NEJMoa2101544 PMC8220996

[12] Bergwerk M, Gonen T, Lustig Y, et al. COVID-19 breakthrough infections in vaccinated health care workers. N Engl J Med. 2021;385(16):1474–1484. 34320281 10.1056/NEJMoa2109072 PMC8362591

[13] Brosh-Nissimov T, Orenbuch-Harroch E, Chowers M, et al. BNT162b2 vaccine breakthrough: clinical characteristics of 152 fully vaccinated hospitalized COVID-19 patients in Israel. Clin Microbiol Infect. 2021;27(11):1652–1657. 34245907 10.1016/j.cmi.2021.06.036 PMC8261136

[14] Lipsitch M, Krammer F, Regev-Yochay G, et al. SARS-CoV-2 breakthrough infections in vaccinated individuals: measurement, causes and impact. Nat Rev Immunol. 2022;22(1):57–65. 34876702 10.1038/s41577-021-00662-4 PMC8649989

[15] Tenforde MW, Self WH, Adams K, et al. Association between mRNA vaccination and COVID-19 hospitalization and disease severity. Jama. 2021;326(20):2043–2054. 34734975 10.1001/jama.2021.19499 PMC8569602

[16] García LF. Immune response, inflammation, and the clinical spectrum of COVID-19. Front Immunol. 2020;11:1441. 32612615 10.3389/fimmu.2020.01441 PMC7308593

[17] Mouliou DS, Gourgoulianis KI. COVID-19 ‘asymptomatic’ patients: an old wives’ tale. Expert Rev Respir Med. 2022;16(4):399–407. 35041796 10.1080/17476348.2022.2030224

[18] Han X, Fan Y, Alwalid O, et al. Six-month follow-up chest CT findings after severe COVID-19 pneumonia. Radiology. 2021;299(1):E177–e186. 33497317 10.1148/radiol.2021203153 PMC7841877

[19] Li X, Shen C, Wang L, et al. Pulmonary fibrosis and its related factors in discharged patients with new corona virus pneumonia: a cohort study. Respir Res. 2021;22(1):203. 34243776 10.1186/s12931-021-01798-6 PMC8267229

[20] Mylvaganam RJ, Bailey JI, Sznajder JI, et al. Recovering from a pandemic: pulmonary fibrosis after SARS-CoV-2 infection. Eur Respir Rev. 2021;30(162):210194. 34911696 10.1183/16000617.0194-2021 PMC8674935

[21] Kim SB, Ryoo S, Huh K, et al. Revised Korean Society of Infectious diseases/national evidence-based healthcarea Collaborating Agency guidelines on the treatment of patients with COVID-19. Infect Chemother. 2021;53(1):166–219. 34409790 10.3947/ic.2021.0303 PMC8032920

[22] Haas EJ, Angulo FJ, McLaughlin JM, et al. Impact and effectiveness of mRNA BNT162b2 vaccine against SARS-CoV-2 infections and COVID-19 cases, hospitalisations, and deaths following a nationwide vaccination campaign in Israel: an observational study using national surveillance data. Lancet. 2021;397(10287):1819–1829. 33964222 10.1016/S0140-6736(21)00947-8 PMC8099315

[23] Bernal J L, Andrews N, Gower C, et al. Effectiveness of the pfizer-BioNTech and Oxford-AstraZeneca vaccines on COVID-19 related symptoms, hospital admissions, and mortality in older adults in England: test negative case-control study. Br Med J. 2021;373: n1088. 33985964 10.1136/bmj.n1088 PMC8116636

[24] Butt AA, Omer SB, Yan P, et al. SARS-CoV-2 vaccine effectiveness in a high-risk national population in a real-world setting. Ann Intern Med. 2021;174(10):1404–1408. 34280332 10.7326/M21-1577 PMC8381771

[25] Chemaitelly H, Tang P, Hasan MR, et al. Waning of BNT162b2 vaccine protection against SARS-CoV-2 infection in Qatar. N Engl J Med. 2021;385(24):e83. 34614327 10.1056/NEJMoa2114114 PMC8522799

[26] Katikireddi SV, Cerqueira-Silva T, Vasileiou E, et al. Two-dose ChAdOx1 nCoV-19 vaccine protection against COVID-19 hospital admissions and deaths over time: a retrospective, population-based cohort study in Scotland and Brazil. Lancet. 2022;399(10319):25–35. 34942103 10.1016/S0140-6736(21)02754-9 PMC8687670

[27] Tartof SY, Slezak JM, Fischer H, et al. Effectiveness of mRNA BNT162b2 COVID-19 vaccine up to 6 months in a large integrated health system in the USA: a retrospective cohort study. Lancet. 2021;398(10309):1407–1416. 34619098 10.1016/S0140-6736(21)02183-8 PMC8489881

[28] Chen N, Zhou M, Dong X, et al. Epidemiological and clinical characteristics of 99 cases of 2019 novel coronavirus pneumonia in wuhan, China: a descriptive study. Lancet. 2020;395(10223):507–513. 32007143 10.1016/S0140-6736(20)30211-7 PMC7135076

[29] Carfì A, Bernabei R, Landi F. Persistent symptoms in patients after acute COVID-19. Jama. 2020;324(6):603–605. 32644129 10.1001/jama.2020.12603 PMC7349096

[30] Zhao YM, Shang YM, Song WB, et al. Follow-up study of the pulmonary function and related physiological characteristics of COVID-19 survivors three months after recovery. EClinicalMedicine. 2020;25:100463. 32838236 10.1016/j.eclinm.2020.100463 PMC7361108

[31] Huang Y, Tan C, Wu J, et al. Impact of coronavirus disease 2019 on pulmonary function in early convalescence phase. Respir Res. 2020;21(1):163. 32600344 10.1186/s12931-020-01429-6 PMC7323373

[32] Shah AS, Wong AW, Hague CJ, et al. A prospective study of 12-week respiratory outcomes in COVID-19-related hospitalisations. Thorax. 2021;76(4):402–404. 33273023 10.1136/thoraxjnl-2020-216308

[33] Regev-Yochay G, Amit S, Bergwerk M, et al. Decreased infectivity following BNT162b2 vaccination: A prospective cohort study in Israel. Lancet Reg Health Eur. 2021;7:100150. 34250518 10.1016/j.lanepe.2021.100150 PMC8261633

[34] Naaber P, Tserel L, Kangro K, et al. Dynamics of antibody response to BNT162b2 vaccine after six months: a longitudinal prospective study. Lancet Reg Health Eur. 2021;10:100208. 34514454 10.1016/j.lanepe.2021.100208 PMC8418937

[35] Doria-Rose N, Suthar MS, Makowski M, et al. Antibody persistence through 6 months after the second dose of mRNA-1273 vaccine for COVID-19. N Engl J Med. 2021;384(23):2259–2261. 33822494 10.1056/NEJMc2103916 PMC8524784

[36] Berlin DA, Gulick RM, Martinez FJ. Severe COVID-19. N Engl J Med. 2020;383(25):2451–2460. 32412710 10.1056/NEJMcp2009575

[37] Schmidt AL, Labaki C, Hsu CY, et al. COVID-19 vaccination and breakthrough infections in patients with cancer. Ann Oncol. 2022;33(3):340–346. 34958894 10.1016/j.annonc.2021.12.006 PMC8704021

[38] Papagoras C, Fragoulis GE, Zioga N, et al. Better outcomes of COVID-19 in vaccinated compared to unvaccinated patients with systemic rheumatic diseases. Ann Rheum Dis. 2021;annrheumdis-2021-221539. 10.1136/annrheumdis-2021-221539 34758975

[39] Grasselli G, Zangrillo A, Zanella A, et al. Baseline characteristics and outcomes of 1591 patients infected with SARS-CoV-2 admitted to ICUs of the lombardy region, Italy. Jama. 2020;323(16):1574–1581. 32250385 10.1001/jama.2020.5394 PMC7136855

[40] Centre for Disease Prevention and Control. Underlying medical conditions associated with high risk for severe COVID-19: information for healthcare providers. Available from: https://www.cdc.gov/coronavirus/2019-ncov/need-extra-precautions/people-with-medical-conditions.html (accessed 11 February 2020).

[41] Ryu S, Kim D, Lim JS, et al. Serial interval and transmission dynamics during SARS-CoV-2 delta variant predominance, South Korea. Emerg Infect Dis. 2022;28(2):407–410. 34906289 10.3201/eid2802.211774 PMC8798673

[42] CDC data tracker. Variant proportions. 2022. Available from: https://covid.cdc.gov/covid-data-tracker/#variant-proportions (accessed 28 Jaunary 2022).

[43] European Centre for Disease Prevention and Control. Weekly epidemiological update: Omicron variant of concern (VOC) – week 1 (data as of 7 January 2022) EU/EEA. 2022. Available from: https://www.ecdc.europa.eu/en/news-events/weekly-epidemiological-update-omicron-variant-concern-voc-week-1-data-7-january-2022 (accessed 29 January 2022).

[44] Gruell H, Vanshylla K, Tober-Lau P, et al. mRNA booster immunization elicits potent neutralizing serum activity against the SARS-CoV-2 Omicron variant. Nat Med. 2022;28(3):477–480. 35046572 10.1038/s41591-021-01676-0 PMC8767537

[45] Cheng SMS, Mok CKP, Leung YWY, et al. Neutralizing antibodies against the SARS-CoV-2 Omicron variant BA.1 following homologous and heterologous CoronaVac or BNT162b2 vaccination. Nat Med. 2022;28(3):486–489. 35051989 10.1038/s41591-022-01704-7 PMC8940714

