# Supplementary material for: Impact of prior vaccination on clinical outcomes of patients with COVID-19
Source: Emerg Microbes Infect. 2022 May 23;11(1):1316–24. doi: 10.1080/22221751.2022.2069516 (PMC9132471; doi:10.1080/22221751.2022.2069516)

**Supplementary File**

**Vaccinated patients are less likely to have** **coronavirus disease 2019 pneumonia and require supplemental oxygen for breakthrough infections**

Woo Jung Seo,^1^ Jiyeon Kang,^1^ Hyung Koo Kang,^1^ So Hee Park,^1^ Hyeon-Kyoung Koo,^1^ Hye Kyeong Park^1^, Sung-Soon Lee,^1^ Je Eun Song,^2^ Yee Gyung Kwak,^2^ Jieun Kang,^1^

^1^Division of Pulmonary and Critical Care Medicine, Department of Internal Medicine, Ilsan Paik Hospital, Inje University College of Medicine, Goyang, Republic of Korea

^2^Division of Infectious Diseases, Department of Internal Medicine, Ilsan Paik Hospital, Inje University College of Medicine, Goyang, Republic of Korea

| Severity categories | Definition |
| --- | --- |
| Mild | Individuals who have any of the various signs and symptoms of COVID-19 (e.g., fever, cough, sore throat, malaise, headache, muscle pain, nausea, vomiting, diarrhoea, loss of taste and smell) but who do not have shortness of breath, dyspnoea, or abnormal chest imaging. |
| Moderate | Individuals who show evidence of lower respiratory disease during clinical assessment or imaging and who have an oxygen saturation (SpO2) ≥94% on room air at sea level. |
| Severe | Individuals who have SpO2 <94% on room air at sea level, a ratio of arterial partial pressure of oxygen to fraction of inspired oxygen (PaO2/FiO2) <300 mm Hg, a respiratory rate >30 breaths/min, or lung infiltrates >50%. |
| Critical | Individuals who have respiratory failure, septic shock, and/or multiple organ dysfunction. |

**Supplementary Table 1. Severity of disease categories**

The severity categories are based on the National Institutes of Health, US criteria.

**Supplementary Table 2. COVID-19 presentation and clinical course of study patients according to the vaccine type**

|  | **ChAdOx1 nCoV-19**  **(n = 100)** | **BNT162b2**  **(n = 98)** | **mRNA-1273**  **(n = 1)** | **Ad26.COV2.S**  **(n = 5)** |
| --- | --- | --- | --- | --- |
| Asymptomatic infection, (%) | 12 (12.0) | 16 (16.3) | 0 (0.0) | 0 (0.0) |
| Hypoxia^*^ at admission, (%) | 12 (12.0) | 8 (8.2) | 0 (0.0) | 6 (100.0) |
| Pneumonia, (%) | 43 (43.0) | 30 (30.6) | 0 (0.0) | 3 (50.0) |
| Supplemental oxygen requirement | 16 (16.0) | 15 (15.3) | 0 (0.0) | 1 (16.7) |
| Respiratory failure | 5 (5.0) | 1 (1.0) | 0 (0.0) | 1 (16.7) |
| Transfer, (%) | 5 (5.0) | 1 (1.0) | 0 (0.0) | 1 (16.7) |
| Treatment, (%) |  |  |  |  |
| Remdesivir | 18 (18.0) | 15 (15.3) | 0 (0.0) | 1 (16.7) |
| Corticosteroid | 23 (23.0) | 21 (21.4) | 0 (0.0) | 1 (16.7) |
| Tocilizumab | 3 (3.0) | 0 (0.0) | 0 (0.0) | 0 (0.0) |
| Regdanvimab | 65 (65.0) | 63 (64.3) | 0 (0.0) | 3 (50.0) |
| Time from symptom onset to discharge, days | 10.0 [10.0;12.0] | 10.0 [9.0;11.0] | 10.0 | 8.5 [7.8;10.0] |

Data are presented as number (%) or median [interquartile range].

^*^Hypoxia was defined as oxygen saturation <94% on room air at sea level.

Abbreviation: COVID-19, coronavirus disease of 2019.

**Supplementary Table 3. Effects of time from the second dose of vaccination to admission on respiratory outcomes**

|  | **OR (95% CI)** | **p-value** |
| --- | --- | --- |
| Hypoxia at admission | 1.009 (1.000–1.019) | 0.061 |
| Pneumonia | 1.005 (0.999–1.011) | 0.097 |
| Supplemental oxygen requirement | 1.009 (1.001–1.017) | 0.022 |
| Respiratory failure | 1.002 (0.986–1.017) | 0.830 |

Abbreviations; OR, odds ratio; CI, confidence interval.

**Supplementary Table 4. Sensitivity analysis of the baseline characteristics of patients according to the vaccination status**

|  | **Vaccinated**  **(n = 190)** | **Unvaccinated**  **(n = 140)** | **p-value** |
| --- | --- | --- | --- |
| Age, years | 66.4 ± 14.4 | 48.2 ± 17.5 | <0.001 |
| Sex |  |  | 0.578 |
| Male | 95 (50.0) | 65 (46.4) |  |
| Female | 95 (50.0) | 75 (53.6) |  |
| BMI, kg/m^2^ | 25.2 ± 3.9 | 25.4 ± 5.1 | 0.662 |
| PCR gene Ct value | 17.0 [13.7;20.4] | 17.5 [14.6;21.1] | 0.472 |
| Co-morbidities |  |  |  |
| Hypertension | 99 (52.1) | 33 (23.6) | <0.001 |
| Diabetes | 40 (21.1) | 19 (13.6) | 0.083 |
| Cardiovascular disease | 33 (17.7) | 20 (14.6) | 0.544 |
| Chronic lung disease | 15 (7.9) | 5 (3.6) | 0.160 |
| Chronic kidney disease | 8 (4.2) | 5 (3.6) | >0.999 |
| Chronic liver disease | 3 (1.6) | 8 (5.7) | 0.059 |
| Solid organ transplantation | 2 (1.1) | 2 (1.4) | >0.999 |
| Rheumatic disorders | 3 (1.6) | 6 (4.3) | 0.176 |
| Cancer | 13 (6.8) | 9 (6.4) | >0.999 |
| Obesity (BMI>30) | 30 (16.5) | 25 (18.5) | 0.655 |

Data are presented as number (%), median [interquartile range], or mean ± standard deviation.

Abbreviations: BMI, body mass index; PCR, polymerase chain reaction; Ct, cycle threshold.

**Supplementary Table 5. Sensitivity analysis of COVID-19 presentation and clinical course of study patients**

|  | **Vaccinated**  **(n = 190)** | **Unvaccinated**  **(n = 140)** | **p-value** |
| --- | --- | --- | --- |
| Asymptomatic infection, (%) | 23 (12.1) | 7 (5.0) | 0.032 |
| Hypoxia^*^ at admission, (%) | 17 (8.9) | 29 (20.7) | 0.003 |
| Pneumonia, (%) | 69 (36.3) | 100 (71.4) | <0.001 |
| Supplemental oxygen requirement | 29 (15.3) | 49 (35.0) | <0.001 |
| Respiratory failure | 7 (3.7) | 11 (7.9) | 0.140 |
| Transfer, (%) | 7 (3.7) | 10 (7.1) | 0.208 |
| Treatment, (%) |  |  |  |
| Remdesivir | 30 (15.8) | 46 (32.9) | <0.001 |
| Corticosteroid | 40 (21.1) | 56 (40.0) | <0.001 |
| Tocilizumab | 3 (1.6) | 5 (3.6) | 0.291 |
| Regdanvimab | 122 (64.2) | 63 (45.0) | 0.001 |
| Time from symptom onset to discharge, days | 10 [9;11] | 12 [10;14] | <0.001 |

Data are presented as number (%) or median [interquartile range].

^*^Hypoxia was defined as oxygen saturation <94% on room air at sea level.

Abbreviation: COVID-19, coronavirus disease of 2019.

**Supplementary Figure 1. Time interval to admission after the second dose of vaccination according to the type of vaccine.**


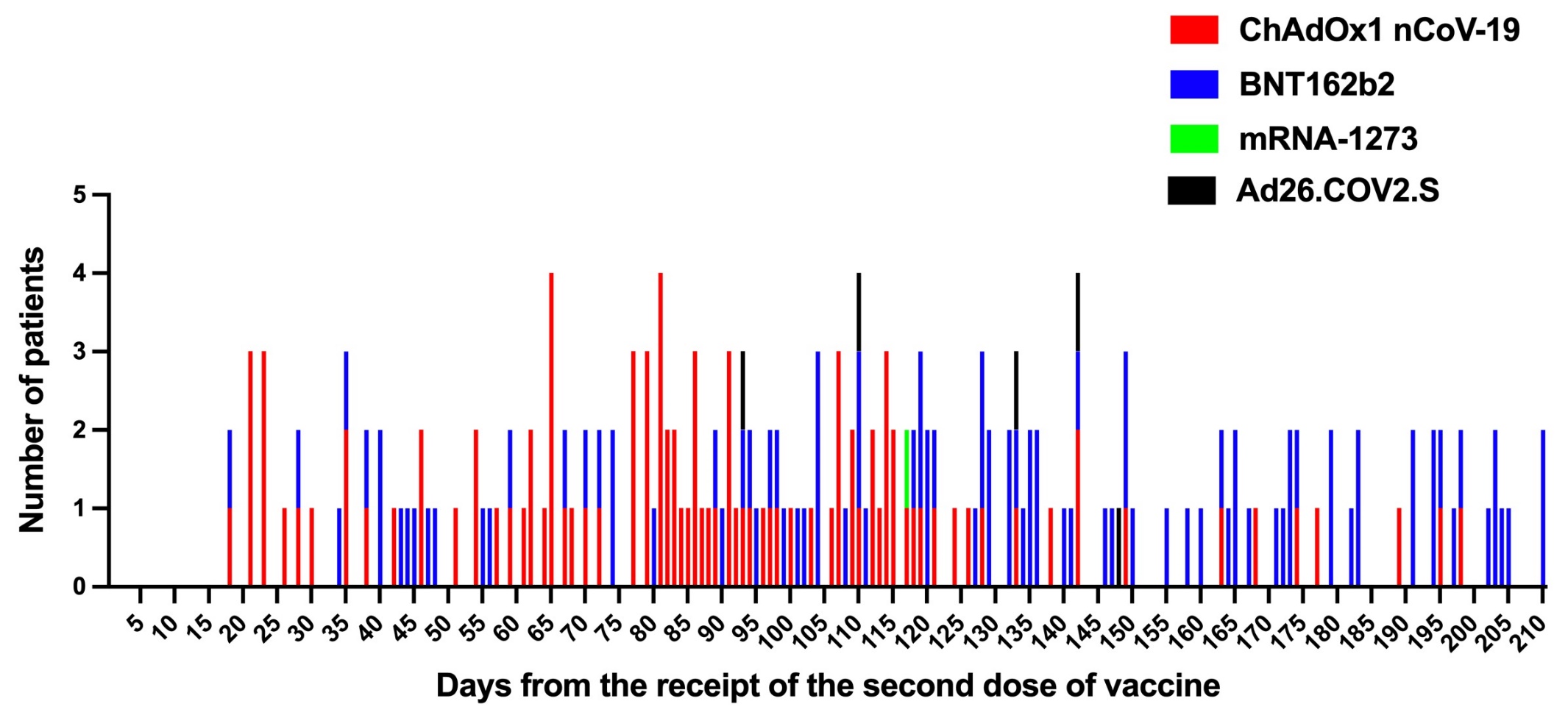

Supplement: Supplemental Material [file TEMI_A_2069516_SM2268.docx]
